# Programme costs of longer and shorter tuberculosis drug regimens and drug import: a modelling study for Karakalpakstan, Uzbekistan

**DOI:** 10.1183/23120541.00622-2021

**Published:** 2022-03-21

**Authors:** Stefan Kohler, Norman Sitali, Jay Achar, Nicolas Paul

**Affiliations:** 1 Charité – Universitätsmedizin Berlin, corporate member of Freie Universität Berlin and Humboldt-Universität zu Berlin, Institute of Social Medicine, Epidemiology and Health Economics, Berlin, Germany; 2 Heidelberg University, Faculty of Medicine and University Hospital, Heidelberg Institute of Global Health, Heidelberg, Germany; 3 Médecins Sans Frontières, Berlin, Germany; 4 Dept of Global Public Health, Karolinska Institutet, Stockholm, Sweden

## Abstract

**Background:**

The introduction of new and often shorter tuberculosis (TB) drug regimens affects the cost of TB programmes.

**Methods:**

We modelled drug purchase and import costs for 20-month, 9-month and 4- to 6-month TB drug regimens based on 2016–2020 treatment numbers from a TB programme in Karakalpakstan, Uzbekistan, and 2021 Global Drug Facility prices.

**Results:**

On average, 2225±374 (±sd) people per year started TB treatment, 30±2.1% of whom were diagnosed with drug-resistant forms of TB. Transitioning from a 6-month to a 4-month drug-susceptible (DS)-TB drug regimen increased the TB programme's annual DS-TB drug cost from USD 65±10 K to USD 357±56 K (p<0.001) and its drug import cost from USD 6.4±1.0 K to USD 9.3±1.4 K (p=0.008). Transitioning from a 20-month all-oral multidrug-resistant (MDR)-TB drug regimen to a 9-month MDR-TB drug regimen with an injectable antibiotic decreased the TB programme's annual MDR-TB drug cost from USD 1336±265 K to USD 266±53 K (p<0.001) and had no significant effect on the drug import cost (USD 28±5.5 K *versus* USD 27±5.4 K; p=0.88). Purchasing (USD 577±114 K) and importing (USD 3.0±0.59 K) the 6-month all-oral MDR-TB drug regimen cost more than procuring the 9-month MDR-TB drug regimen but less than the 20-month all-oral MDR-TB drug regimen (both p<0.01).

**Conclusion:**

Introducing new and shorter TB drug regimens could increase the cost of TB programmes with low drug resistance rates and decrease the cost of TB programmes with high drug resistance rates.

## Abstract 2

**Introduction of new TB drug regimens can increase or decrease programme expenses for drug purchase and import. Modelling programme costs of different drug regimens can inform financial planning and help prepare TB programmes for changes in treatment.** https://bit.ly/3r8coRj

## Introduction

With 1.3 million fatalities in 2020, tuberculosis (TB) is among the top causes of death worldwide [1]. The standard treatment of drug-susceptible (DS)-TB requires taking the four first-line TB drugs rifampicin, isoniazid, pyrazinamide and ethambutol for an intensive treatment phase of 2 months, followed by taking rifampicin and isoniazid for a continuation phase of 4 months [2]. The treatment of multidrug-resistant (MDR)-TB with resistance to rifampicin and isoniazid can require taking up to seven antibiotics per day. Conventional MDR-TB treatment, which is no longer recommended by the World Health Organization (WHO), included the use of an injectable antibiotic and lasted up to 2 years [3]. Newer and often shorter treatment regimens with new or repurposed TB drugs have been evaluated and have entered clinical practice. For DS-TB, the WHO endorsed a 4-month drug regimen in June 2021 as an alternative to the standard 6-month treatment [4, 5]. For MDR-TB, the WHO currently recommends the use of an all-oral, 20-month MDR-TB drug regimen [6]. The use of all-oral 6- to 11-month regimens is conditionally recommended [6], and all-oral MDR-TB drug regimens of 26 weeks and shorter have been and are being evaluated in clinical trials (*e.g.* Nix TB trial, NeXT Study, ZeNix TB trial, a BPaMZ regimen trial, TB PRACTECAL trial or endTB-Q trial [7–12]).

TB drug regimen choices affect treatment effectiveness, treatment tolerability, quality of life on treatment and expenses for TB drugs. The programme cost for TB drugs includes expenses for drug purchase and often import costs for international drug procurement. In many instances, TB drugs are procured from the Global Drug Facility that supplies drugs to TB programmes worldwide [13, 14]. The purchase of TB drugs is a major contributor to TB treatment costs. In studies of TB programmes in Peru, Estonia, Russia and the Philippines, TB drugs contributed 35%, 19%, 26% and 46%, respectively, to the costs of conventional MDR-TB treatment [15–17]. In another study of a TB programme in Nigeria, drugs contributed 6.5–12.6% to the costs of conventional MDR-TB treatment, 8.8–15.6% to the costs of 9–12-month MDR-TB treatment with a regimen including an injectable antibiotic and 25.8% to the costs of 9–12-month all-oral MDR-TB treatment [18]. For a 6-month DS-TB treatment, a systematic review found that the purchase of TB drugs contributes 14.2% to the TB treatment costs in lower-middle-income countries and 19% in low-income countries [19].

Previous studies have estimated and compared the costs of purchasing and/or importing DS-TB and MDR-TB drug regimens [20–22]. These studies found substantial cost differences between TB drug regimens. To our knowledge, the study at hand is the first to assess the impact of the adaption of new DS-TB and MDR-TB drug regimens on the total drug procurement cost of a TB programme. Specifically, we modelled the costs of TB drug purchase and import for longer and new, shorter TB drug regimens for a TB programme in Karakalpakstan, Uzbekistan. As our model includes drug purchase and import costs, we also assessed the impact of a transport cost increase, as observed, for instance, during the coronavirus disease 2019 (COVID-19) pandemic, on the programme cost for drug procurement.

## Methods

### Study setting

Uzbekistan is listed by the WHO as one of 30 high MDR-TB burden countries [1]. In Karakalpakstan, a republic in Uzbekistan's northwest with a population of 1.9 million people in 2019 [23], the TB prevalence [25] and MDR-TB rate among newly diagnosed TB cases [26] were about twice Uzbekistan's national average in 2013 and 2010/2011, respectively. Médecins Sans Frontières (MSF) has been supporting TB control in Karakalpakstan since 1998 [24]. In addition to standard 6-month DS-TB treatment [2], longer and shorter MDR-TB drug regimens have been used and evaluated in the TB programme [11, 27–30]. The new 4-month DS-TB drug regimen [4, 5] was not offered in 2021. TB drugs and other medical supplies for the programme are regularly imported from an MSF procurement unit in the Netherlands to the TB programme in Karakalpakstan as humanitarian goods using air and land transport [31].

### Study design

We modelled the costs of procuring, *i.e.* purchasing and importing, TB drugs from the perspective of the TB programme in Karakalpakstan. Modelling was based on, first, our previous estimation of drug regimen costs and drug import costs to the TB programme [21, 31], and second, the number of people who started treatment for DS-TB or drug-resistant TB in Karakalpakstan between 2016 and 2020 [32] (see supplementary table S1). We compared the drug procurement costs for different model scenarios, in which the TB programme provides either longer or shorter DS-TB and MDR-TB drug regimens. Following the terminology used in the 2020 WHO guidelines for treatment of drug-resistant TB [6], we refer to MDR-TB drug regimens of at least 18 months as longer drug regimens and to MDR-TB drug regimens with a duration of <12 months as shorter drug regimens. In addition, we refer to TB drug regimens that require 6 months of treatment or less as short regimens.

### Model scenarios

For DS-TB, we modelled treatment with two short, all-oral DS-TB drug regimens. For drug-resistant TB, we modelled treatment with two longer MDR-TB drug regimens, one shorter, and one short MDR-TB drug regimen. In the DS-TB reference scenario 1, people with DS-TB are treated with a standard 6-month drug regimen with fixed-dose combinations of two to four oral antibiotics. In DS-TB scenario 2, people with DS-TB are treated with a new 4-month (17 weeks) regimen, which has been endorsed by the WHO in June 2021 [4]. In the MDR-TB reference scenario 1, people with MDR-TB are treated with a longer, 20-month all-oral drug regimen. In MDR-TB scenario 2, people with MDR-TB are treated with a shorter, 9-month MDR-TB drug regimen with an injectable antibiotic. In MDR-TB scenario 3, people with MDR-TB are treated with a short, 6-month all-oral drug regimen that has been conditionally recommended by the WHO in June 2020 [6]. In MDR-TB scenario 4, people with MDR-TB are treated with a conventional 20-month MDR-TB drug regimen, which includes an injectable antibiotic and is no longer recommended by the WHO (table 1) [3, 6].

**TABLE 1:** Purchase costs and import costs of longer and shorter tuberculosis (TB) drug regimens for a TB programme in Karakalpakstan, Uzbekistan

**TB drug regimen used for treatment (scenario)**	**Tablets and injections per regimen**	**Import weight per regimen kg**	**Drug cost per regimen USD**	**Import cost per regimen USD**	**Import cost (% of drug cost)**
**Drug-susceptible TB treatment**					
2 HRZE/4 HR (reference scenario 1)	730	0.79	43	4.17	9.8
2 Rpt-H-Z-Mfx/2 Rpt-H-Mfx (scenario 2)	1358	1.1	233	6.04	2.6
**Multidrug-resistant TB treatment**					
20 Bdq-Lfx-Lzd-Cfz-Cs (reference scenario 1)	4824	7.8	1977	41	2.1
4 Km-Mfx-Pto-Cfz-Z-H^h^-E/5 Z-E-Mfx-Pto-Cfz (scenario 2)	3650	7.6	393	40	10.3
6 Bdq-Pa-Lzd (scenario 3)	748	0.84	855	4.45	0.52
8 Z-Km-Lfx-PAS-Pto-Cs/12 Z-Lfx-PAS-Pto-Cs (scenario 4)	9368	27	2442	144	5.9

Months and drugs for intensive phase/continuation phase of treatment. The exact duration of the 2 Rpt-H-Z-Mfx/2 Rpt-H-Mfx regimen is 8 weeks/9 weeks. The TB drug regimens included in the model represent the least costly regimens and lowest treatment durations from a larger set of DS-TB and MDR-TB drug regimens described in supplementary table S2. HRZE/HR: fixed-dose combination of four/two oral antibiotics; 2 Rpt-H-Z-Mfx: 2 months of single doses of the antibiotics Rpt, H, Z and Mfx; Bdq: bedaquiline 100 mg; Cs: cycloserine 250 mg; Cfz: clofazimine 100 mg; E: ethambutol 400 mg; H: isoniazid 300 mg; H^h^: high-dose isoniazid 600 mg; Km: kanamycin 1000 mg (injectable antibiotic); Lfx: levofloxacin 250 mg; Lzd: linezolid 600 mg; Mfx: moxifloxacin 400 mg; Pa: pretomanid 200 mg; PAS: PAS sodium 4 g; Pto: prothionamide 250 mg; R: rifampicin 300 mg; Rpt: rifapentine 150 mg; Z: pyrazinamide 400 mg. *Data sources:* Kohler *et al*. [21, 31].

### Model parameters

#### Number of people treated for TB

Treatment numbers in the model are based on the number of people starting TB treatment in the TB programme in Karakalpakstan. As a simplification, we assume that all people with drug-resistant TB receive the same MDR-TB drug regimen. Further, we assume that drug procurement costs to the programme occur in the year in which TB treatment is started and that all people with TB receive drug dosing for an adult weighing 60 kg.

The number of people starting TB treatment in the TB programme in Karakalpakstan has been steadily declining over the past years, with an expedited decline during the COVID-19 pandemic. In 2016, 2645 people with TB started treatment as compared to 2130 and 1662 in 2019 and 2020, respectively. Between 27% and 33% (mean±sd 30±2.1%) of the people commencing TB treatment between 2016 and 2020 were diagnosed with drug-resistant forms of TB. Treatment numbers by TB type are provided in supplementary table S1.

#### TB drug regimen procurement costs

The model's drug regimen costs and import costs are based on costing studies which we previously conducted for the TB programme in Karakalpakstan [21, 31]. In a prior analysis of TB drug regimen costs [21], we extracted drug quantities required for various longer, shorter and short drug regimens from TB programme guidelines for Karakalpakstan and Uzbekistan, WHO guidelines and TB trial protocols. To estimate the procurement cost, we multiplied the drug quantities required by a regimen with their respective purchase prices and import costs. TB drug prices were extracted from the Global Drug Facility Medicines Catalog (October 2021) [33]. Import cost estimates of individual TB drugs stemmed from our previous micro-costing study that assessed air freight, customs and land freight costs of shipping medical supplies from the MSF procurement unit in Amsterdam, the Netherlands, to the TB programme's central storage in Nukus, Karakalpakstan, in 2016 [31]. The drug regimen import costs used in the modelling were adjusted for inflation and changes in exchange rates between 2016 and 2021 [21] (table 1).

For the DS-TB drug regimen with a fixed-dose combination of antibiotics over 6 months, we estimated a purchase cost of USD 43, to which import to Karakalpakstan adds USD 4.17 at 2021 prices. For the 4-month DS-TB drug regimen, we estimated a purchase cost of USD 233, to which import adds USD 6.04. Purchasing drugs for the 20-month all-oral MDR-TB regimen costs USD 1977, to which import adds USD 41. The 9-month MDR-TB drug regimen with an injectable antibiotic costs USD 393 to purchase and USD 40 to import. The 6-month all-oral MDR-TB drug regimen costs USD 791 to purchase and USD 2.72 to import. In comparison, a conventional 20-month MDR-TB drug regimen with an injectable antibiotic cost USD 2442 to purchase and USD 144 to import.

### Programme cost modelling and data analysis

Annual drug procurement costs for the TB programme were modelled for the years 2016–2020 by multiplying the number of people commencing DS-TB and MDR-TB treatment in a year with the procurement costs of the drug regimens assumed in the model scenarios. 2016–2020 cost means were calculated and compared using two-sample t-tests with unequal variances. Percentage cost differences were assessed for being significantly different from zero using one-sample t-tests. To assess the impact of import cost fluctuations on the TB programme cost, we modelled a ±50% change in air freight charges. Analyses were performed in Stata 15.1 SE (StataCorp, College Station, TX, USA) and costs are reported in 2021 US dollars (USD).

### Ethical considerations

No ethical approval was sought as this modelling study used secondary data.

## Results

### Programme costs of drug-susceptible TB drug regimens

**FIGURE 1:**
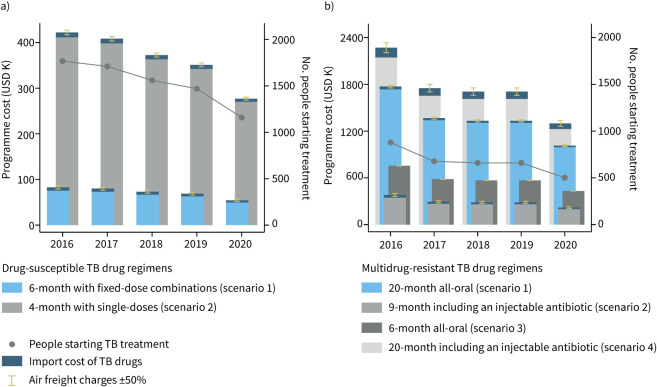
Programme costs of longer and shorter tuberculosis (TB) drug regimens. a) Drug-susceptible TB treatment; b) multidrug-resistant TB treatment. y-axes for programme costs have different scales. Programme costs for the years 2016–2020 are based on the drug regimen choice, the annual number of people starting TB treatment in a TB program in Karakalpakstan, Uzbekistan, and TB drug prices from the October 2021 Global Drug Facility Medicines Catalog. The TB drugs and dosing used in the drug regimens are summarised in table 1. K: thousand.

In the DS-TB reference scenario 1, people with DS-TB are treated with a 6-month drug regimen with fixed-dose combinations. The TB programme's annual cost (±sd) of purchasing DS-TB drugs is USD 65±10 K per year. The cost of importing DS-TB drugs is USD 6.4±1.0 K per year. In DS-TB scenario 2, people with DS-TB are treated with a 4-month regimen. The TB programme's annual cost of purchasing DS-TB drugs increases to USD 357±56 K (+USD 291±25 K; p<0.001) in comparison to the DS-TB reference scenario. The annual cost of importing DS-TB drugs increases to USD 9.3±1.4 K (+USD 2.9±0.79 K; p=0.008) (table 2 and figure 1a).

**TABLE 2:** Programme costs of longer and shorter tuberculosis (TB) drug regimens

**TB drug regimen used for treatment (scenario)**	**Total procurement cost USD K (±sd)**	**Drug cost USD K (±sd)**	**Import cost USD K (±sd)**
**Drug-susceptible TB treatment**			
6-month (reference scenario 1)	72±11	65±10	6.4±1.0
4-month (scenario 2)	366±57	357±56	9.3±1.4
Δ to reference scenario	294±26***	357±56	2.9±0.79**
Δ to reference scenario (%)^#^	410	446	45
Cost range	72–366	65–357	6.4–9.3
**Multidrug-resistant TB treatment**			
20-month all-oral (reference scenario 1)	1363±270	1336±265	28±5.5
9-month including an injectable antibiotic (scenario 2)	293±58	266±53	27±5.4
Δ to reference scenario	−1070±123.5***	−1070±121***	−0.54±3.5
Δ to reference scenario (%)^#^	−78.5	−80	−1.9
6-month all-oral (scenario 3)	580±115	577±114	3.0±0.59
Δ to reference scenario	−783±131**	−758±129**	−25±2.5***
Δ to reference scenario (%)^#^	−57	−57	−89
Δ to 9-month scenario	287±58**	312±56**	−24±2.4***
Δ to 9-month scenario (%)^#^	98	117	−89
20-month including an injectable antibiotic (scenario 4)	1747±346	1649±327	97±19
Δ to reference scenario	383±196	314±188	70±9.0***
Δ to reference scenario (%)^#^	28	23	250
Cost range	293–1363 [1747]	266–1336 [1649]	3.0–28 [97]

The TB drugs and dosing of the regimens are described in table 1. A breakdown of the import costs into air freight, land freight and customs-related costs is provided in supplementary table S3. K: thousand; Δ: difference. ^#^: as the percentage difference does not vary between years, no standard deviation and p-value are reported. [ ]: no longer recommended drug regimen. *: p<0.05; **: p<0.01; ***: p<0.001.

### Programme costs of multidrug-resistant TB drug regimens

In the MDR-TB reference scenario 1, people with MDR-TB are treated with a 20-month all-oral drug regimen. The TB programme's annual cost of purchasing MDR-TB drugs is USD 1336±265 K. The annual cost of importing MDR-TB drugs is USD 28±5.5 K per year (figure 1b).

In MDR-TB scenario 2, people with MDR-TB are treated with a 9-month MDR-TB drug regimen with an injectable antibiotic. By adapting the 9-month MDR-TB drug regimen, the TB programme's annual cost of purchasing MDR-TB drugs decreases to USD 266±53 K (−USD 1070±121 K; p<0.001) in comparison to the MDR-TB reference scenario. The annual cost of importing MDR-TB drugs (USD 27±5.4 K, −USD 0.54±3.5 K; p=0.88) is similar to the MDR-TB reference scenario.

In MDR-TB scenario 3, people with MDR-TB are treated with a 6-month all-oral MDR-TB drug regimen. By adapting the 6-month MDR-TB drug regimen, the TB programme's annual cost of purchasing MDR-TB drugs decreases to USD 577±114 K (−USD 758±129 K; p=0.002) and the annual cost of importing MDR-TB drugs decreases to USD 3.0±0.59 K (−USD 25±2.5; p<0.001) in comparison to the MDR-TB reference scenario.

The procurement of the 6-month MDR-TB drug regimen is costlier for the TB programme than the procurement of the 9-month MDR-TB drug regimen with an injectable antibiotic (+USD 287±58 K, p=0.003) despite import cost savings (−USD 24±2.4 K; p<0.001) of the 6-month regimen in comparison to the 9-month regimen. The procurement of either the 9-month or 6-month MDR-TB drug regimen costs the TB programme less than the procurement of the 20-month all-oral MDR-TB drug regimen in the reference scenario (both p<0.01).

In MDR-TB scenario 4, people with MDR-TB are treated with a 20-month MDR-TB drug regimen with an injectable antibiotic. The annual cost of procuring MDR-TB drugs in this scenario (USD 1747±346 K, +USD 383±196; p=0.089) is similar to the cost of procuring drugs in the 20-month all-oral MDR-TB reference scenario. The drug import cost, however, is substantially higher for the 20-month MDR-TB drug regimen with an injectable antibiotic (USD 97±19 K, +USD 70±9.0 K; p<0.001) in comparison to the 20-month all-oral MDR-TB reference scenario.

### Total drug procurement cost of the TB programme

The total drug procurement cost of the TB programme depends on the number of people on TB treatment, the share of drug resistance among people on TB treatment, and the combination of DS-TB and MDR-TB drug regimens provided. Purchasing drugs for 6-month DS-TB treatment and 20-month all-oral MDR-TB treatment (reference combination) costs the TB programme USD 1401±274 K per year, to which drug import adds USD 34±6.4 K per year (table 3).

**TABLE 3:** Programme costs of tuberculosis (TB) drug regimen combinations

**Drug regimens used for drug-susceptible and multidrug-resistant TB treatment (combination)**	**Total procurement cost USD K (±sd)**	**Drug cost USD K (±sd)**	**Import cost USD K (±sd)**
**6-month and 20-month all-oral (reference combination)**	1435±280	1401±274	34±6.4
**6-month and 9-month including an injectable antibiotic (least costly combination)**	365±68	331±62	34±6.3
Δ to reference combination	−1070±129***	−1070±125***	−0.54±4.0
Δ to reference combination (%)	−75±0.31***	−76±0.30***	−1.6±0.025***
**4-month and 20-month all-oral (costliest combination)**	1730±322	1692±315	37±6.8
Δ to reference combination	294±191	291±187	2.9±4.2
Δ to reference combination (%)	21±1.6***	21±1.6***	8.4±0.57***
**4-month and 6-month all-oral (shortest combination)**	947±168	934±166	12±2.0
Δ to reference combination	−489±146*	−467±143*	−22±3.0***
Δ to reference combination (%)	−34±1.8***	−33±1.9***	−64±1.7***
**6-month and 20-month including an injectable antibiotic (phased-out combination)**	1819±356	1715±336	104±20
Δ to reference combination	383±203	314±194	70±9.5***
Δ to reference combination (%)	27±0.11***	22±0.087***	203±3.2***
**Cost range**	365–1730 [1819]	331–1692 [1715]	12–37 [104]

The TB drugs and dosing of the regimens are described in table 1. A breakdown of the import costs into air freight, land freight and customs-related costs is provided in supplementary table S3. K: thousand; Δ difference. [ ]: no longer recommended drug regimen. *: p<0.05; **: p<0.01; ***: p<0.001.

If the TB programme combines the least costly DS-TB and MDR-TB drug regimens assessed, *i.e.* the 6-month DS-TB drug regimen and the 9-month MDR-TB drug regimen with an injectable antibiotic, its drug purchasing cost falls to USD 331±62 K (−USD 1070±125 K; p<0.001) in comparison to the reference combination of drug regimens. The TB programme's import cost remains similar to the reference combination when the least costly combination of DS-TB and MDR-TB drug regimens is used for treatment (USD 34±6.3 K, −USD 0.54±4.0 K; p=0.90). If the TB programme uses the costliest combination of the assessed drug regimens, *i.e.* the 4-month DS-TB drug regimen and the 20-month all-oral MDR-TB drug regimen, neither its cost for drug purchase (USD 1692±315 K, +USD 291±187 K; p=0.16) nor its cost for drug import (USD 37±6.8 K, +USD 2.9±4.2 K; p=0.51) increase significantly in comparison to the reference combination. If the TB programme provides the shortest of the assessed DS-TB and MDR-TB drug regimens, *i.e.* the 4-month DS-TB drug regimen and the 6-month MDR-TB drug regimen, the programme costs for purchasing and importing TB drugs fall to USD 934±166 K (−USD 467±143 K; p=0.015) and USD 12±2.0 (−USD 22±3.0 K; p<0.001), respectively, in comparison to the reference combination of TB drug regimens.

Providing the costliest combination of DS-TB and MDR-TB drug regimens, *i.e.* the 4-month DS-TB drug regimen and the 20-month all-oral MDR-TB drug regimen (USD 1730±322 K), rather than the least costly regimen combination, *i.e.* the 6-month DS-TB drug regimen and the 9-month MDR-TB drug regimen with an injectable antibiotic (USD 365±68 K), almost quintuples (4.7±0.0059+374±0.59%) the drug procurement cost of the TB programme. The TB programme's import cost is highest when the phased-out 20-month MDR-TB drug regimen is used together with the 4-month DS-TB drug regimen. When the DS-TB and MDR-TB drug regimens with the hightest import costs are combined, then the programme's import cost for TB drugs is 211±2.6% higher than of the import cost of the reference combination and 1032±57% higher than of the import cost of the least-costly-to-import combination of 6-month all-oral drug regimens for DS-TB and MDR-TB treatment (supplementary table S3).

### Programme vulnerability to import cost fluctuations

Air freight was responsible for 95% of the modelled import costs to the TB programme based on our previous import cost assessment [31]. The error bars in figure 1 indicate how the import cost of the TB programme increases (decreases) when the average cost of air freight changes from USD 4.87 per kg weight imported to USD 7.31 (USD 2.44) per kg, *i.e.* by ±50%. During the COVID-19 pandemic, air and sea freight charges to many destinations increased by a similar or even higher magnitude [34, 35]. A 50% rise in air freight charges increases the TB programme's annual drug import cost from USD 34±6.4 K to USD 51±9.5 K (+USD 16±5.1 K; p=0.013) in the reference combination of drug regimens, *i.e.* when 6-month DS-TB and 20-month all-oral MDR-TB drug regimens are used for treatment in the TB programme. If the TB programme used the combination of drug regimens that is most costly to import, *i.e.* the 4-month DS-TB drug regimen and the conventional 20-month MDR-TB drug regimen with an injectable antibiotic, a 50% rise in air freight charges would increase the TB programme's annual drug import cost from USD 107±21 K to USD 157±30 K (+USD 51±16 K; p=0.015). Comparing the height of the lines that show a ±50% change in air freight charges in figure 1 across scenarios illustrates how a higher import cost renders a TB programme more vulnerable to a transport cost increase.

## Discussion

Despite an increase in spending on TB and a decrease in the TB incidence globally between 2000 and 2017, many TB programmes continue to face a funding gap, especially in low-income and lower-middle-income countries [1, 36]. The procurement cost of TB drug regimens can therefore affect how many people receive TB treatment. MSF estimated, for example, that only 11% of the eligible people with TB have received bedaquiline worldwide between 2015 and 2019, related to financial constraints that restrict access to newer, more expensive TB drugs such as bedaquiline, delamanid and pretomanid [20]. Modelling programme costs for drug procurement can identify financial needs and potential cost savings, and thereby helps to prepare TB programmes for new TB drug regimens.

This modelling study compared the drug procurement cost of a TB programme in Karakalpakstan, Uzbekistan, for different scenarios in which 20-month, 9-month, 6-month and 4-month drug regimens were used for TB treatment. The assessed TB drug regimens include the latest WHO treatment recommendations. The modelling was based on context-specific information obtained in prior bottom-up costing studies, 2016–2020 treatment numbers and 2021 TB drug prices from the Global Drug Facility. Therefore, the presented findings can inform the TB programme in Karakalpakstan and, to some degree, also TB programmes elsewhere about the possible budget impact of introducing some of the latest and shortest TB drug regimens.

In the reference scenario of the model, we found that drug purchase for the currently recommended 6-month DS-TB treatment and the 20-month all-oral MDR-TB treatment generates annual costs of USD 1401±274 K for the TB programme, to which drug import adds USD 34±6.4 K per year. The total drug procurement expenses of USD 1435±280 K per year for this combination of DS-TB and MDR-TB drug regimens correspond to 23.2±1.9% of MSF's average annual expenditures for activities in Uzbekistan between 2016 and 2020 (compare supplementary tables S1 and S3). The use of a 4-month rifapentine-based DS-TB drug regimen, which was recommended by the WHO in June 2021 [4], increased drug procurement costs in the model when used instead of the present 6-month standard DS-TB drug regimen. This increase in drug procurement cost is driven by the present higher cost of rifapentine and its availability in the form of 150 mg tablets only [21]. The restricted choice in rifapentine formulations requires to take eight tablets per day to reach the target dose of 1200 mg for an adult weighing 55–75 kg. The resulting need to import more tablets for the new 4-month DS-TB drug regimen in comparison to a standard DS-TB drug regimen with fixed-dose combinations increased the weight of the imported drugs and, thus, the import cost of the TB programme in our model. Furthermore, use of either a 9-month MDR-TB drug regimen with an injectable antibiotic or a 6-month all-oral MDR-TB drug regimen reduced the drug procurement cost when compared to the currently WHO-recommended 20-month all-oral MDR-TB drug regimen [6]. The latter reduction in MDR-TB drug procurement cost can be attributed to a shorter treatment duration, the need for fewer tablets or injectables and the use of cheaper TB drugs [21]. Given the relatively high prevalence of drug-resistant strains of TB in Karakalpakstan, the combined introduction of 4-month DS-TB treatment and either 9-month or 6-month MDR-TB treatment reduced the overall drug procurement cost of the TB programme in the assessed scenarios, as higher DS-TB treatment costs were outweighed by cost savings in shorter than 20-month MDR-TB treatment.

The magnitude of the contribution of drug import costs to the total drug procurement cost varied between scenarios. The TB programme's import costs for an all-oral 20-month MDR-TB drug regimen and a 9-month injectable-based MDR-TB drug regimen were similar as cost savings from importing a lower quantity of TB drugs were offset by a heavier weight of the injectable antibiotic, which is imported as solvable powder in a relatively heavy glass vial. The adoption of an all-oral 6-month MDR-TB drug regimen, in turn, reduced the drug import cost of the TB programme compared to the longer MDR-TB drug regimens assessed. Of all DS-TB and MDR-TB regimens studied in the model, the by far highest import cost for the TB programme was associated with the no longer WHO-recommended 20-month MDR-TB drug regimen with an injectable antibiotic. For TB programmes that still provide conventional MDR-TB treatment, transitioning to shorter and/or all-oral MDR-TB drug regimens seems therefore highly likely to reduce the cost of importing TB drugs.

High import cost relative to the drug procurement cost can make a TB programme vulnerable to import cost shocks (*e.g.* a steep increase in air or sea freight charges as observed during the COVID-19 pandemic [34, 35]). As TB programmes transition to shorter all-oral MDR-TB drug regimens, some TB programmes could experience import cost reductions and, thus, become less vulnerable to import cost fluctuations. Not only the import of fewer drugs, but also the import of less heavy drugs can result in import cost savings (*e.g.* use of fixed-dose combinations, higher-dosed tablets or lighter tablets). With respect to the new 4-month DS-TB drug regimen, the WHO recently called for manufacturing rifapentine in higher-dosed tablets and fixed-dose combinations [37].

The strengths of this modelling study include its timely analysis of new drug regimens for TB treatment, the use of drug prices from the Global Drug Facility, through which many TB programmes procure TB drugs, and the model parameterisation with data from the TB programme in Karakalpakstan. Limitations of the study include, firstly, that we assumed that all people with drug-resistant -TB in the TB programme in Karakalpakstan were treated with the same MDR-TB drug regimen. Programme data indicate that 66±10% of the people with drug-resistant TB starting treatment between 2016 and 2020 had MDR-TB, whereas 9.1±2.0% had extensively drug-resistant TB, which requires a more individualised treatment approach than MDR-TB, and 24±12% were diagnosed with other forms of drug resistance [32]. Secondly, the modelling inherited limitations of our previous study in which we generated the unit import costs underlying the import cost estimation of the study at hand [31]. The inherited limitations include that import cost estimates were based on the assessment of costs and unit weights of only one major shipment to the TB programme in 2016. Further, import costs were generated based on catalogued and imputed unit weights, which might have been measured or estimated with low precision. Thirdly, this study modelled the impact of the transition to shorter TB drug regimens on drug procurement costs but did not assess effects of the adoption of shorter TB drug regimens on other programme costs, such as administration [38], side-effect treatment, contact tracing or treatment monitoring. Fourthly, we used only Global Drug Facility prices as inputs to our model, but the TB programme in Karakalpakstan also procures TB drugs from other sources such as MSF. Conversely, using exclusively Global Drug Facility prices instead of programme-specific drug costs might make our results more relevant for other settings, as globally a large share of quality-assured TB drugs is procured through the Global Drug Facility [14]. Finally, by construction of the model, the statistical comparisons of the calculated cost differences are based on variation in the number of people starting TB treatment over time only. While yearly drug prices and import costs could have been modelled, holding these costs constant allowed to focus on the programme cost variation caused by drug regimen choices and fluctuations in treatment numbers.

### Conclusion

Modelling the purchase and import costs of longer and shorter DS-TB and MDR-TB drug regimens for a TB program in Karakalpakstan, Uzbekistan, suggests that the use of shorter TB drug regimens in favour of longer regimens can increase or decrease the drug procurement cost of a TB programme. The introduction of a new 4-month DS-TB drug regimen increased the drug procurement cost in comparison to the standard 6-month DS-TB treatment in our model. The introduction of either a 9-month MDR-TB drug regimen with an injectable antibiotic or a 6-month all-oral MDR-TB drug regimen decreased the TB programme's drug procurement cost in our model in comparison to the use of a 20-month all-oral drug regimen. Whether the combined introduction of new and shorter DS-TB and MDR-TB drug regimens increased or decreased the total drug procurement cost of the TB programme at 2021 prices depended on the new and replaced TB drug regimens as well as on the prevalence of drug-resistant TB in the programme setting.

## Supplementary material

10.1183/23120541.00622-2021.Supp1 **Please note:** supplementary material is not edited by the Editorial Office, and is uploaded as it has been supplied by the author.
Supplementary material 00622-2021.SUPPLEMENT
Supplementary material ERJOR-00622-2021.R1 - TB PROGRAM COST_SUPPLEMENTARY MATERIAL
