# Supplementary material for: Programme costs of longer and shorter tuberculosis drug regimens and drug import: a modelling study for Karakalpakstan, Uzbekistan
Source: ERJ Open Res. 2022 Mar 21;8(1):00622-2021. doi: 10.1183/23120541.00622-2021 (PMC8943289; doi:10.1183/23120541.00622-2021)
Supplement: Supplementary file 1 [file 00622-2021.SUPPLEMENT.pdf]

Supplementary material

Table S1: Number of people starting tuberculosis (TB) treatment in a TB programme in Karakalpakstan, Uzbekistan

Year	Staff	Program cost (EUR M)	Number of people starting TB treatment					DR-TB share (%)
			Total	DS-TB	DR-TB	MDR-TB	XDR-TB	
2016	248	9.8	2645	1767	878	433	63	33
2017	264	9	2466	1710	677	524	79	27
2018	271	8.6	2220	1560	660	450	70	30
2019	301	8.3	2130	1470	660	465	58	31
2020	310	7	1662	1160	502	344	36	30
2016–20 mean	279±26	8.5±1.0	2225±374	1533±240	675±134	443±65	61±16	30.3±2.1

±SD. DS = drug-susceptible, DR = drug-resistant, MDR = multidrug-resistant, XDR = extensively drug-resistant.
Data sources: Médecins Sans Frontières activity reports [1] and personal communication.

Table S2: Purchase costs and import costs of longer and shorter tuberculosis (TB) drug regimens for a TB programme in Karakalpakstan, Uzbekistan

TB drug regimen used for treatment (scenario—if included in modelling study)	Tablets and injections per day	Tablets and injections per regimen	Import weight per regimen (kg)	Drug cost per regimen (USD)	Import cost per regimen (USD)	Import cost (% of drug cost)	Total cost per month (USD)
**Drug-susceptible (DS)-TB treatment**							
6-month DS-TB drug regimen[#]							
2 HRZE/4 HR fixed-dose combination (scenario 1)	4	730	0.79	43	4.17	9.8	7.11
2 H-R-Z-E/4 H-R	3–10	973	0.94	64	5.28	8.2	11
4-month (17-weeks) DS-TB drug regimen[*]							
8w Rpt-H-Z-Mfx/9w Rpt-H-Mfx (scenario 2)	10–13	1358	1.1	233	6.04	2.6	59
**Multidrug-resistant (MDR)-TB treatment**							
20-month all-oral MDR-TB drug regimens[†]							
20 Bdq-Lfx-Lzd-Cfz-Cs (scenario 1)	7–11	4824	7.8	1977	41	2.1	99
20 Bdq-Dlm-Lzd-Cfz-Cs	7–11	4824	6.4	5000	34	0.68	250
9–11-month MDR-TB drug regimens with an injectable antibiotic[‡]							
4–6 Km-Mfx-Pto-Cfz-Z-H^h-E/5 Z-E-Mfx-Pto-Cfz (scenario 2)	12–15	3650–4563	7.6–11	393–506	40–56	10.3–11.1	44–46
4–6 Cm-Mfx-Pto-Cfz-Z-H^h-E/5 Z-E-Mfx-Pto-Cfz	12–15	3650–4563	8.1–11	585–793	43–59	7.3–7.5	65–72
6–11-month all-oral MDR-TB drug regimens							
4–6 Bdq (6 m)-Lfx-Cfz-Z-E-H^h-Eto/5 Lfx-Cfz-Z-E[§]	6–18	3729–4580	3.5–4.4	668–738	19–23	2.8–3.1	67–74
6–9 Bdq-Pa-Lzd (scenario 3)[¶]	3–7	748–1100	0.84–1.2	855–1243	4.45–6.63	0.52–0.53	138–142
20–24-month MDR-TB drug regimens with an injectable antibiotic[‖]							
8 Z-Km-Lfx-PAS-Pto-Cs/12–16 Z-Lfx-PAS-Pto-Cs (scenario 4)	15–16	9368–11 193	27–31	2442–2895	144–164	5.7–5.9	121–122
8 Z-Km-Mfx-PAS-Pto-Cs/12–16 Z-Mfx-PAS-Pto-Cs	14–15	8760–10 463	26–29	2484–2946	136–155	5.3–5.5	123–124
8 Z-Cm-Lfx-PAS-Pto-Cs/12–16 Z-Lfx-PAS-Pto-Cs	15–16	9368–11 193	28–32	2825–3278	149–169	5.1–5.3	137–141
8 Z-Cm-Mfx-PAS-Pto-Cs/12–16 Z-Mfx-PAS-Pto-Cs	14–15	8760–10 463	27–30	2867–3329	141–159	4.8–4.9	139–143

Months and drugs for intensive phase/continuation phase of TB treatment. w = weeks when regimen duration is not reported in months. Import weight includes drugs, drug packaging and cargo packaging. Cargo packaging added, on average, 8.9% to the import weight. [#] Standard drug regimen [2]. [*] Recently endorsed by the WHO [3] based on the TBTC Study 31 [4]. [†] Used in Uzbekistan for people with medium or high risk of failing MDR/rifampicin-resistant TB treatment [5, 6]. [‡] Tested in a 2013–15 prospective trial in the TB programme in Karakalpakstan [7]. [§] The shorter all-oral bedaquiline-containing MDR/rifampicin-resistant TB regimen was recommended by WHO in 2020 [6]. [¶] The BPaL regimen was found effective in the multi-centre Nix-TB trial in South Africa [8] and is WHO recommended under operational research conditions [6]. [‖] Conventional MDR-TB drug regimens as recommended in 2015 TB treatment guidelines in Karakalpakstan [9]. Drug acronyms and formulations are described in the caption of **Table 1** and in Kohler et al. [10]. *Data source:* Kohler et al. [10, 11].

Table S3: Programme costs of longer and shorter tuberculosis (TB) drug regimens and drug regimen combinations

Year	Total procurement cost (USD K)	Drug cost (USD K)	Import cost (USD K)			
			Total	Air freight	Customs-related	Land freight
**Drug regimens used for drug-susceptible (DS) TB treatment**						
6-month DS-TB drug regimen (scenario 1)						
2016	83	75	7.4	7.0	0.046	0.33
2017	80	73	7.1	6.8	0.044	0.32
2018	73	67	6.5	6.2	0.041	0.29
2019	69	63	6.1	5.8	0.038	0.28
2020	54	49	4.8	4.6	0.030	0.22
2016–20 mean	72±11	65±10	6.4±1.0	6.1±0.95	0.040±0.0062	0.29±0.045
4-month DS-TB drug regimen (scenario 2)						
2016	422	411	11	10	0.12	0.48
2017	408	398	10	9.8	0.12	0.47
2018	373	363	9	8.9	0.11	0.42
2019	351	342	9	8.4	0.10	0.40
2020	277	270	7	6.6	0.081	0.32
2016–20 mean	366±57	357±56	9.3±1.4	8.7±1.4	0.11±0.017	0.42±0.065
Δ to reference scenario	294±26***	291±25***	2.9±0.79**	2.7±0.74**	0.067±0.0080***	0.13±0.036**
Δ to reference scenario (%)[#]	410	446	45	44	169	44
**Drug regimens used for multidrug-resistant (MDR) TB treatment**						
20-month all-oral MDR-TB drug regimen (scenario 1)						
2016	1772	1736	36	34	0.22	1.6
2017	1367	1339	28	26	0.17	1.3
2018	1332	1305	27	26	0.16	1.2
2019	1332	1305	27	26	0.16	1.2
2020	1013	993	21	20	0.12	0.94
2016–20 mean	1363±270	1336±265	28±5.5	26±5.2	0.17±0.033	1.3±0.25

Year	Total procurement cost (USD K)	Drug cost (USD K)	Import cost (USD K)			
			Total	Air freight	Customs-related	Land freight
9-month MDR-TB drug regimen with an injectable antibiotic (scenario 2)						
2016	381	345	36	34	0.25	1.6
2017	294	266	27	26	0.19	1.2
2018	286	260	27	25	0.19	1.2
2019	286	260	27	25	0.19	1.2
2020	218	197	20	19	0.14	0.92
2016–20 mean	293±58	266±53	27±5.4	26±5.1	0.19±0.038	1.2±0.24
Δ to reference scenario	−1070±124***	−1070±121***	−0.54±3.5	−0.54±3.3	0.024±0.023	−0.026±0.16
Δ to reference scenario (%)#	−79	−80	−1.9	−2.0	14	−2.0
6-month all-oral MDR-TB drug regimen (scenario 3)						
2016	754	751	3.9	3.7	0.034	0.18
2017	582	579	3.0	2.8	0.026	0.14
2018	567	564	2.9	2.8	0.025	0.13
2019	567	564	2.9	2.8	0.025	0.13
2020	431	429	2.2	2.1	0.019	0.10
2016–20 mean	580±115	577±114	3.0±0.59	2.8±0.56	0.026±0.0052	0.14±0.027
Δ to reference scenario	−783±131**	−758±129**	−25±2.5***	−24±2.4***	−0.14±0.015***	−1.1±0.11***
Δ to reference scenario (%)#	−57	−57	−89	−89	−84	−89
Δ to 9-month regimen	287±58**	312±56**	−24±2.4***	−23±2.3***	−0.17±0.017***	−1.1±0.11***
Δ to 9-month regimen (%)#	98	117	−89	−89	−86	−89
20-month MDR-TB drug regimen with an injectable antibiotic (scenario 4)						
2016	2271	2144	127	120	0.57	5.7
2017	1751	1653	98	93	0.44	4.4
2018	1707	1612	95	90	0.43	4.3
2019	1707	1612	95	90	0.43	4.3
2020	1298	1226	72	69	0.33	3.3
2016–20 mean	1747±346	1649±327	97±19	93±18	0.44±0.087	4.4±0.87
Δ to reference scenario	383±196	314±188	70±9.0***	66±8.5***	0.27±0.041**	3.2±0.41***
Δ to reference scenario (%)#	28	23	250	250	163	250

Year	Total procurement cost (USD K)	Drug cost (USD K)	Import cost (USD K)			
			Total	Air freight	Customs-related	Land freight
**Drug regimen combinations used for drug-susceptible (DS) & drug-resistant (DR) TB treatment**						
6-month DS-TB drug regimen & 20-month all-oral MDR-TB drug regimen (reference combination)						
2016–20 mean	1435±280	1401±274	34±6.4	32±6.1	0.21±0.039	1.6±0.29
6-month DS-TB drug regimen & 9-month MDR-TB drug regimen with an injectable antibiotic (least costly combination)						
2016–20 mean	365±68	331±62	34±6.3	32±6.0	0.23±0.043	1.5±0.29
Δ to reference combination	−1070±129***	−1070±125***	−0.54±4.0	−0.54±3.8	0.024±0.026	−0.026±0.18
Δ to reference combination (%)	−75±0.31***	−76±0.30***	−1.6±0.025***	−1.7±0.026***	11±0.18***	−1.7±0.026***
4-month DS-TB drug regimen & 20-month all-oral MDR-TB drug regimen (costliest combination)						
2016–20 mean	1730±322	1692±315	37±6.8	35±6.5	0.28±0.049	1.7±0.31
Δ to reference combination	294±191	291±187	2.9±4.2	2.7±4.0	0.067±0.028*	0.13±0.19
Δ to reference combination (%)	21±1.6***	21±1.6***	8.4±0.57***	8.3±0.56***	33±2.2***	8.3±0.56***
Δ to least costly combination	1365±147***	1361±143***	3.4±4.2	3.2±3.9	0.044±0.029	0.15±0.19
Δ to least costly combination (%)	374±0.59***	411±0.57***	10±0.55***	10±0.54***	19±2.2***	10±0.54***
4-month DS-TB drug regimen & 6-month all-oral MDR-TB drug regimen (shortest combination)						
2016–20 mean	947±168	934±166	12±2.0	12±1.9	0.13±0.021	0.55±0.08978
Δ to reference combination	−489±146*	−467±143*	−22±3.0***	-21±2.8***	-0.074±0.020**	-1.0±0.14***
Δ to reference combination (%)	−34±1.8***	−33±1.9***	−64±1.7***	-64±1.7***	-36±3.3***	-64±1.7***
Δ to least costly combination	582±81***	603±79***	−21±3.0***	-20±2.8***	-0.098±0.022**	-0.97±0.13***
Δ to least costly combination (%)	160±4.1***	183±4.3***	−63±1.7***	-64±1.7***	-42±3.0***	-64±1.7***
6-month DS-TB drug regimen & 20-month MDR-TB drug regimen with an injectable antibiotic (phased-out combination)						
2016–20 mean	1819±356	1715±336	104±20	99±19	0.48±0.092	4.7±0.91
Δ to reference combination	383±203	314±194	70±9.5***	66±9.0***	0.27±0.045**	3.2±0.43***
Δ to reference combination (%)	27±0.11***	22±0.087***	203±3.2***	203±3.2***	131±2.1***	203±3.2***
Δ to least costly combination	1454±162***	1384±153***	70±9.5***	67±9.0***	0.25±0.046**	3.2±0.43***
Δ to least costly combination (%)	398±6.5***	417±6.9***	208±3.3***	208±3.3***	108±1.5***	208±3.3***
4-month DS-TB drug regimen & 9-month MDR-TB drug regimen with an injectable antibiotic (other combination)						
2016–20 mean	659±112	623±105	37±6.7	35±6.4	0.30±0.053	1.7±0.30
Δ to reference combination	−776±135**	−778±131**	2.3±4.2	2.1±3.9	0.091±0.029*	0.10±0.19
Δ to reference combination (%)	−54±1.9***	−55±1.9***	6.9±0.59***	6.6±0.58***	44±2.0***	6.6±0.58***
Δ to least costly combination	294±59**	291.5±55**	2.9±4.1	2.7±3.9	0.067±0.031	0.13±0.19
Δ to least costly combination (%)	81±5.4***	89±5.9***	8.6±0.575***	8.4±0.57***	29±2.0***	8.4±0.57***

Year	Total procurement cost (USD K)	Drug cost (USD K)	Import cost (USD K)			
			Total	Air freight	Customs-related	Land freight
6-month DS-TB drug regimen & 6-month all-oral MDR-TB drug regimen (least-costly-to-import combination)						
2016–20 mean	652±125	643±123	9.4±1.5	8.9±1.5	0.066±0.011	0.42±0.070
Δ to reference combination	−783±137**	−758±134**	−25±3.0***	−24±2.8***	−0.14±0.018***	−1.1±0.13***
Δ to reference combination (%)	−55±0.23***	−54±0.21***	−72±1.1***	−72±1.1***	−68±1.1***	−72±1.1***
4-month DS-TB drug regimen & 20-month MDR-TB drug regimen with an injectable antibiotic (costliest-to-import combination)						
2016–20 mean	2113±397	2006±377	107±218	101±20	0.55±0.10	4.8±0.93
Δ to reference combination	678±217*	605±208*	72±9.6***	69±9.2***	0.34±0.049***	3.3±0.44***
Δ to reference combination (%)	47±1.5***	43±1.6***	211±2.6***	212±2.6***	164±0.080***	212±2.6***
Δ to least-costly-to-import combination	1461±186***	1363±177***	97±9.2***	92±8.8***	0.48±0.046***	4.4±0.42***
Δ to least-costly-to-import combination (%)	224±1.7***	212±2.0***	1032±57***	1035±57***	731±29***	1035±57***

K = thousand. ±SD. Δ = difference in 2016–20 means. [#] As the percentage difference does not vary between years, no standard deviation and p-value are reported. Air freight costs were due to international shipping by plane from Amsterdam airport to Tashkent airport. Customs-related import costs include costs of a customs agent assisting with the declaration of the imported medical supplies and costs for storage at Tashkent airport during the declaration process. Land freight costs were due to national transport by truck from Tashkent airport to a central storage in Karakalpakstan's capital Nukus. The TB drugs and dosing used in these regimens are provided in **Table 1**.

References

1. Médecins Sans Frontières. *International Activity Reports 2016–2020: Uzbekistan*. www.msf.org/international-activity-report-2016/uzbekistan, www.msf.org/international-activity-report-2017/uzbekistan, www.msf.org/international-activity-report-2018/uzbekistan, www.msf.org/international-activity-report-2019/uzbekistan and www.msf.org/uzbekistan Date last accessed: 23 January 2022.
2. World Health Organization. *Guidelines for Treatment of Drug-Susceptible Tuberculosis and Patient Care*. Geneva, World Health Organization, 2017.
3. World Health Organization. *Treatment of Drug-Susceptible Tuberculosis: Rapid Communication*. Geneva, World Health Organization, 2021.
4. Dorman, S.E., et al., *Four-month rifapentine regimens with or without moxifloxacin for tuberculosis.* The New England Journal of Medicine, 2021. 384(18): p. 1705-1718.
5. Safaev, K., et al., *Trends, characteristics and treatment outcomes of patients with drug-resistant tuberculosis in Uzbekistan: 2013–2018.* International Journal of Environmental Research and Public Health, 2021. 18(9): p. 4663.
6. World Health Organization. *WHO Operational Handbook on Tuberculosis. Module 4: Treatment. Drug-Resistant Tuberculosis Treatment*. Geneva, World Health Organization, 2020.
7. du Cros, P., et al., *Outcomes with a shorter multidrug-resistant tuberculosis regimen from Karakalpakstan, Uzbekistan.* ERJ Open Research, 2021. 7(1): p. 00537-2020.
8. Conradie, F., et al., *Treatment of highly drug-resistant pulmonary tuberculosis.* The New England Journal of Medicine, 2020. 382(10): p. 893-902.
9. Ministry of Health of the Republic of Karakalpakstan, Médecins Sans Frontières. *Clinical Guidelines on Comprehensive TB Treatment for Drug-Sensitive and Drug-Resistant Tuberculosis, Karakalpakstan, Uzbekistan*. Nukus and Tashkent, Ministry of Health of the Republic of Karakalpakstan and Médecins Sans Frontières, 2015.
10. Kohler, S., et al., *The contribution of drug import to the cost of tuberculosis treatment: a cost analysis of longer, shorter and short drug regimens for Karakalpakstan, Uzbekistan*. Heidelberg University, Heidelberg Institute of Global Health, 2022. mimeo.
11. Kohler, S., N. Sitali, and N. Paul, *A framework for assessing import costs of medical supplies and results for a tuberculosis program in Karakalpakstan, Uzbekistan.* Health Data Science, 2021.
