# Supplementary material for: Programme costs of longer and shorter tuberculosis drug regimens and drug import: a modelling study for Karakalpakstan, Uzbekistan
Source: ERJ Open Res. 2022 Mar 21;8(1):00622-2021. doi: 10.1183/23120541.00622-2021 (PMC8943289; doi:10.1183/23120541.00622-2021)
Supplement: Supplementary file 2 [file 00622-2021.SUPPLEMENTARY.docx]

**Supplementary materials**

**Table S1:** Number of people starting tuberculosis (TB) treatment in the TB program in Karakalpakstan, Uzbekistan

| **Year** | **Staff** | **Program**  **cost (mil. €)** | **Number of people starting TB treatment** | | | | | **DR-TB share (%)** |
| --- | --- | --- | --- | --- | --- | --- | --- | --- |
|  |  |  | **Total** | **DS-TB** | **DR-TB** | **MDR-TB** | **XDR-TB** |  |
| 2016 | 248 | 9.8 | 2,645 | 1,767 | 878 | 433 | 63 | 33 |
| 2017 | 264 | 9.0 | 2,466 | 1,710 | 677 | 524 | 79 | 27 |
| 2018 | 271 | 8.6 | 2,220 | 1,560 | 660 | 450 | 70 | 30 |
| 2019 | 301 | 8.3 | 2,130 | 1,470 | 660 | 465 | 58 | 31 |
| 2020 | 310 | 7 | 1,662 | 1,160 | 502 | 344 | 36 | 30 |
| 2016–20 | 279 (26) | 8.5 (1.0) | 2,225 (374) | 1,533 (240) | 675 (134) | 443 (65) | 61 (16) | 30.3 (2.1) |

(  ) = standard deviation. TB = tuberculosis. TB = tuberculosis, DS = drug-susceptible, DR = drug-resistant, XDR = extensively drug-resistant. *Data sources:* Médecins Sans Frontières activity reports [26] and personal communication.

**Table S2:** Amount of drugs in a tuberculosis (TB) drug regimen, drug purchase cost, and drug import cost for a TB program in Karakalpakstan, Uzbekistan

| **Drug regimen used for TB treatment (scenario—if included in modelling study)** | **Tablets and injectables per day** | **Tablets and injectables per regimen** | **Import weight per regimen (kg)** | **Drug cost per regimen ($)** | **Import cost per regimen ($)** | **Import cost (% of regimen cost)** | **Total cost per patient month ($)** |
| --- | --- | --- | --- | --- | --- | --- | --- |
|  | | | | | | | |
| **Drug-susceptible (DS)-TB** | | | | | | | |
| ***6-months DS-TB drug regimens^a^*** | | | | | | | |
| 2 HRZE / 4 HR fixed-dose combination (scenario 1) | 4 | 731 | 0.8 | 43 | 4.05 | 9.5 | 7.79 |
| 2 H-R-Z-E / 4 H-R | 3–9.75 | 959 | 0.9 | 64 | 5.05 | 7.9 | 11 |
| ***4-months (17-weeks) DS-TB drug regimen^b^*** | | | | | | | |
| 8w Rpt-H-Z-Mfx / 9w Rpt-H-Mfx (scenario 2) | 13 | 1548 | 1.3 | 237 | 6.74 | 2.8 | 62 |
|  | | | | | | | |
| **Multidrug-resistant (MDR)-TB** | | | | | | | |
| ***20-months all-oral MDR-TB drug regimens^c^*** | | | | | | | |
| 20 Bdq-Lfx-Lzd-Cfz-Cs (scenario 1) | 10.4–13 | 6356 | 8.1 | 2816 | 42 | 1.5 | 143 |
| 20 Bdq-Dlm-Lzd-Cfz-Cs | 8.38–11 | 5139 | 6.8 | 5832 | 35 | 0.6 | 293 |
| ***9–11-months MDR-TB drug regimens with an injectable antibiotic^d^*** | | | | | | | |
| 4–6 Km-Mfx-Pto-Cfz-Z-H^h^-E / 5 Z-E-Mfx-Pto-Cfz (scenario 2) | 13–17 | 3926–5083 | 7.9–14 | 482–637 | 41–72 | 8.4–11.3 | 58–64 |
| 4–6 Cm-Mfx-Pto-Cfz-Z-H^h^-E / 5 Z-E-Mfx-Pto-Cfz | 13–17 | 3926–5083 | 8.3–15 | 668–918 | 43–75 | 6.4–8.2 | 79–90 |
| ***6–11-months all-oral MDR-TB drug regimens*** | | | | | | | |
| 4–6 Bdq(6 m)-Lfx-Cfz-Z-E-H^h^-Eto / 5 Lfx-Cfz-Z-E^e^ | 12–21 | 4101–5220 | 3.9–4.9 | 706–943 | 20–25 | 2.7–2.8 | 81–88 |
| 6–9 Bdq-Pa-Lzd (scenario 3)*^f^* | 4–6 | 654–963 | 0.7–1 | 975–1429 | 3.70–5.49 | 0.4 | 159–163 |
| ***20–24-months MDR-TB drug regimens with an injectable antibiotic^g^*** | | | | | | | |
| 8 Z-Km-Lfx-PAS-Pto-Cs / 12–16 Z-Lfx-PAS-Pto-Cs (scenario 1) | 17–19 | 10592–12906 | 27–35 | 2448–2935 | 140–181 | 5.7–6.2 | 129–130 |
| 8 Z-Cm-Mfx-PAS-Pto-Cs / 12–16 Z-PAS-Pto-Cs | 13–16 | 8401–10227 | 26–33 | 2797–3272 | 133–170 | 4.7–5.2 | 143–146 |
| 8 Z-Cm-Lfx-PAS-Pto-Cs / 12–16 Z-Lfx-PAS-Pto-Cs | 17–19 | 10592–12906 | 28–36 | 2821–3309 | 144–185 | 5.1–5.6 | 146–148 |

Intensive phase / continuation phase of treatment. TB = tuberculosis, DS = drug-susceptible, MDR = multidrug-resistant, w = weeks. Drug acronyms and dosing are described in the caption of **Table 1** and [20]. ^a^Standard drug regimen [2]. ^b^Recently endorsed by the WHO [4] based on the TBTC Study 31 [33]. ^c^ Used for people with medium or high risk of failed treatment in Uzbekistan [5, 34]. ^d^Tested in a 2013–15 prospective trial in the TB program in Karakalpakstan [35]. ^e^For people with MDR/RR-TB without resistance to fluoroquinolones. 9–12-months according to WHO guidelines [5]. ^f^ Effective in a multi-center trial in South Africa [6] and recommended by the WHO under certain operational research conditions [5]. ^g^Recommended in 2015 TB program guidelines [36]. Weight includes drug weight and drug packaging; cargo packaging added, on average, 8.9% to cargo weight for drugs imported to the TB program in Karakalpakstan [20]. *Data sources:* Kohler et al. [20, 25]

**Table S3:** Modelled drug purchase and import costs of a tuberculosis (TB) program in Karakalpakstan, Uzbekistan, for longer and shorter TB drug regimens, 2016–2020

| **Year** | **Total procurement cost ($)** | **Drug cost ($)** | **Import cost ($)** | | | | |
| --- | --- | --- | --- | --- | --- | --- | --- |
|  |  |  | **Total** | **Air freight (% of total)** | **Customs−related (% of total)** | | **Land freight (% of total)** |
|  | | | | | | | |
| **Drug regimen used for drug-susceptible (DS)-TB treatment** | | | | | | | |
| ***6-months DS-TB drug regimen (scenario 1)*** | | | | | | | |
| 2016 | 82,573 | 75,418 | 7,155 | 6,775 (95) | | 56 (0.78) | 324 (4.5) |
| 2017 | 79,909 | 72,985 | 6,924 | 6,556 (95) | | 54 (0.78) | 314 (4.5) |
| 2018 | 72,900 | 66,583 | 6,317 | 5,981 (95) | | 49 (0.78) | 286 (4.5) |
| 2019 | 68,694 | 62,742 | 5,952 | 5,636 (95) | | 46 (0.78) | 270 (4.5) |
| 2020 | 54,207 | 49,510 | 4,697 | 4,448 (95) | | 37 (0.78) | 213 (4.5) |
| 2016–20 mean | 71,657 (11,203) | 65,448 (10,233) | 6,209 (971) | 5,879 (919) | | 48 (7.6) | 281 (44) |
| ***4-months DS-TB drug regimen (scenario 2)*** | | | | | | | |
| 2016 | 430,678 | 418,761 | 11,917 | 11,219 (94) | | 161 (1.4) | 537 (4.5) |
| 2017 | 416,785 | 405,252 | 11,533 | 10,857 (94) | | 156 (1.4) | 520 (4.5) |
| 2018 | 380,225 | 369,704 | 10,521 | 9,905 (94) | | 142 (1.4) | 474 (4.5) |
| 2019 | 358,289 | 348,375 | 9,914 | 9,333 (94) | | 134 (1.4) | 447 (4.5) |
| 2020 | 282,731 | 274,908 | 7,823 | 7,365 (94) | | 106 (1.4) | 353 (4.5) |
| 2016–20 mean | 373,742 (58,434) | 363,400 (56,817) | 10,342 (1,617) | 9,736 (1,522) | | 140 (22) | 466 (73) |
| Δ to reference scenario | 302,085 (26,608) | 297,952 (25,818) | 4,133 (843) | 3,857 (795) | | 91 (10) | 184 (38) |
| P-value | 0.0002 | 0.0002 | 0.002 | 0.002 | | 0.0003 | 0.002 |
| Δ to reference scenario (%)^a^ | 421 | 455 | 66.6 | 65.6 | | 188 | 65.6 |
|  | | | | | | | |
| **Drug regimen used for multidrug-resistant (MDR)-TB treatment** | | | | | | | |
| ***20-months all-oral MDR-TB drug regimen (scenario 1)*** | | | | | | | |
| 2016 | 2,508,865 | 2,472,164 | 36,700 | 34,733 (95) | 305 (0.83) | | 1,663 (4.5) |
| 2017 | 1,934,512 | 1,906,213 | 28,299 | 26,781 (95) | 235 (0.83) | | 1,282 (4.5) |
| 2018 | 1,885,935 | 1,858,347 | 27,588 | 26,109 (95) | 229 (0.83) | | 1,250 (4.5) |
| 2019 | 1,885,935 | 1,858,347 | 27,588 | 26,109 (95) | 229 (0.83) | | 1,250 (4.5) |
| 2020 | 1,434,453 | 1,413,470 | 20,984 | 19,859 (95) | 174 (0.83) | | 951 (4.5) |
| 2016–20 mean | 1,929,940 (382,281) | 1,901,708 (376,689) | 28,232 (5,592) | 26,718 (5,292) | 235 (46) | | 1,279 (253) |
| ***9-months MDR-TB drug regimen with an injectable antibiotic (scenario 2)*** | | | | | | | |
| 2016 | 458,495 | 422,841 | 35,654 | 33,794 (95) | 243 (0.68) | | 1,618 (4.5) |
| 2017 | 353,532 | 326,040 | 27,492 | 26,057 (95) | 187 (0.68) | | 1,247 (4.5) |
| 2018 | 344,655 | 317,853 | 26,802 | 25,403 (95) | 183 (0.68) | | 1,216 (4.5) |
| 2019 | 344,655 | 317,853 | 26,802 | 25,403 (95) | 183 (0.68) | | 1,216 (4.5) |
| 2020 | 262,146 | 241,761 | 20,385 | 19,322 (95) | 139 (0.68) | | 925 (4.5) |
| 2016–20 mean | 352,696 (69,862) | 325,269 (64,429) | 27,427 (5,433) | 25,996 (5,149) | 187 (37) | | 1,244 (247) |
| Δ to reference scenario | −1,577,243 (173,793) | −1,576,439 (170,907) | −805 (3,487) | −722 (3,302) | −48 (27) | | −35 (158) |
| P-value | 0.0006 | 0.0006 | 0.82 | 0.83 | 0.11 | | 0.83 |
| Δ to reference scenario (%)^a^ | −81.7 | −82.9 | −2.85 | −2.70 | −20.4 | | −2.70 |
| ***6-months all-oral MDR-TB drug regimen (scenario 3)*** | | | | | | | |
| 2016 | 859,118 | 855,872 | 3,246 | 3,056 (94) | 44 (1.3) | | 146 (4.5) |
| 2017 | 662,441 | 659,938 | 2,503 | 2,356 (94) | 34 (1.3) | | 113 (4.5) |
| 2018 | 645,807 | 643,367 | 2,440 | 2,297 (94) | 33 (1.3) | | 110 (4.5) |
| 2019 | 645,807 | 643,367 | 2,440 | 2,297 (94) | 33 (1.3) | | 110 (4.5) |
| 2020 | 491,204 | 489,349 | 1,856 | 1,747 (94) | 25 (1.3) | | 84 (4.5) |
| 2016–20 mean | 660,875 (130,906) | 658,378 (130,411) | 2,497 (495) | 2,351 (466) | 34 (6.6) | | 113 (22) |
| Δ to reference scenario | −1,269,064 (180,707) | −1,243,330 (178,270) | −25,735 (2,511) | −24,367 (,2376) | −201 (21) | | −1,167 (114) |
| P-value | 0.001 | 0.001 | 0.0005 | 0.0005 | 0.0005 | | 0.0005 |
| Δ to reference scenario (%)^a^ | −65.8 | −65.4 | −91.2 | −91.2 | −85.7 | | −91.2 |
| Δ to 9-months regimen | 308,179 (66,358) | 333,109 (65,051) | −24,930 (2,440) | −23,645 (2,312) | −153 (17) | | −1,132 (111) |
| P-value | 0.003 | 0.002 | 0.0005 | 0.0005 | 0.0006 | | 0.0005 |
| Δ to 9-months regimen (%)^a^ | 87.4 | 102 | −90.9 | −91.0 | −82.0 | | −91.0 |
| ***20-months MDR-TB drug regimen with an injectable antibiotic (scenario 4)*** | | | | | | | |
| 2016 | 2,271,757 | 2,148,927 | 122,830 | 116,617 (95) | 631 (0.51) | | 5,583 (4.5) |
| 2017 | 1,751,685 | 1,656,974 | 94,711 | 89,920 (95) | 486 (0.51) | | 4,305 (4.5) |
| 2018 | 1,707,699 | 1,615,366 | 92,333 | 87,662 (95) | 474 (0.51) | | 4,197 (4.5) |
| 2019 | 1,707,699 | 1,615,366 | 92,333 | 87,662 (95) | 474 (0.51) | | 4,197 (4.5) |
| 2020 | 1,298,886 | 1,228,657 | 70,229 | 66,676 (95) | 360 (0.51) | | 3,192 (4.5) |
| 2016–20 mean | 1,747,545 (346,152) | 1,653,058 (327,436) | 94,487 (18,716) | 89,707 (17,769) | 485 (96) | | 4,295 (851) |
| Δ to reference scenario | −182,395 (230,634) | −248,650 (223,208) | 66,255 (8,736) | 62,989 (8,292) | 250 (48) | | 3,015 (397) |
| P-value | 0.45 | 0.3 | 0.0008 | 0.0008 | 0.002 | | 0.0008 |
| Δ to reference scenario (%)^a^ | −9.45 | −13.1 | 235 | 236 | 107 | | 236 |
|  | | | | | | | |
| **Combination of drug regimens used for TB treatment (DS-TB & MDR-TB)** | | | | | | | |
| ***6-months & 20-months all oral (reference combination)*** | 2,001,596 (175,387) | 1,967,156 (172,502) | 34,441 (2,890) | 32,597 (2,736) | 283 (24) | | 1,561 (131) |
| ***6-months / 20-months with an injectable antibiotic (phased-out combination)*** | 1,819,202 (159,232) | 1,718,506 (150,478) | 100,696 (8,755) | 95,587 (8311) | 533 (46) | | 4,576 (398) |
| Δ to reference combination | −182,395 (236,887) | −248,650 (228,911) | 66,255 (9,219) | 62,989 (8,749) | 250 (52) | | 3,015 (419) |
| P-value | 0.4636 | 0.3096 | 0.0009 | 0.0009 | 0.0029 | | 0.0009 |
| Δ to reference combination (%) | −9.11 (0.012) | −12.6 (0.016) | 192 (1.29) | 192 (1.30) | 88.4 (0.56) | | 193 (1.30) |
| P-value | < 0.001 | < 0.001 | < 0.001 | < 0.001 | < 0.001 | | < 0.001 |
| Δ to least costly combination | 1,394,849 (163,192) | 1,327,789 (154,035) | 67,060 (9,197) | 63,712 (8730) | 298 (50.0) | | 3050 (419) |
| P-value | 0.0007 | 0.0007 | 0.0009 | 0.0009 | 0.0014 | | 0.0009 |
| Δ to least costly combination (%) | 328 (2.1) | 339 (2.1) | 199 (1.4) | 200 (1.4) | 127 (0.97) | | 200 (1.4) |
| P-value | < 0.001 | < 0.001 | < 0.001 | < 0.001 | < 0.001 | | < 0.001 |
| ***6-months & 9-months with an injectable antibiotic (least costly combination)*** | 424,353 (35,732) | 390,717 (32,913) | 33,636 (2,819) | 31,875 (2672) | 235 (20) | | 1,526 (128) |
| Δ to reference combination | −1,577,243 (178,990) | −1,576,439 (175,614) | −805 (4,038) | −722 (3,824) | −48 (31) | | −35 (183) |
| P-value | 0.0006 | 0.0006 | 0.85 | 0.85 | 0.16 | | 0.85 |
| Δ to reference combination (%) | −78.8 (0.10) | −80.1 (0.099) | −2.33 (0.016) | −2.21 (0.015) | −16.9 (0.11) | | −2.21 (0.015) |
| P-value | < 0.001 | < 0.001 | < 0.001 | < 0.001 | < 0.001 | | < 0.001 |
| ***4-months & 20-months all oral (costliest*** ***combination)*** | 2,303,681 (194,359) | 2,265,108 (191,206) | 38,573 (3,156) | 3,6454 (2,984) | 374 (30) | | 1,745 (143) |
| Δ to reference combination | 302,085 (261,794) | 297,952.5 (257,520) | 4,133 (4,289) | 3,857 (4,048) | 91 (38) | | 185 (194) |
| P-value | 0.28 | 0.28 | 0.36 | 0.37 | 0.045 | | 0.37 |
| Δ to reference combination (%) | 15.2 (0.54) | 15.3 (0.54) | 12.1 (0.37) | 11.9 (0.36) | 32.4 (0.99) | | 11.9 (0.36) |
| P-value | < 0.001 | < 0.001 | < 0.001 | < 0.001 | < 0.001 | | < 0.001 |
| Δ to least costly combination | 1,879,328 (197,616) | 1,874,391 (194,018) | 4,937 (4,232) | 4,579 (4,005) | 139 (36) | | 219 (192) |
| P-value | 0.0005 | 0.0005 | 0.28 | 0.29 | 0.0060 | | 0.29 |
| Δ to least costly combination (%) | 443 (0.13) | 480 (0.15) | 14.7 (0.36) | 14.4 (0.35) | 59.3 (0.99) | | 14.4 (0.35) |
| P-value | < 0.001 | < 0.001 | < 0.001 | < 0.001 | < 0.001 | | < 0.001 |
| ***4-months & 9-months with an injectable antibiotic (other combination)*** | 726,438 (55,645) | 688,669.5 (52,580) | 37,769 (3,085) | 35,732 (2920) | 326 (25.6) | | 1,711 (140) |
| Δ to reference combination | −966,979 (193,814.5) | −945,377 (190,821) | −21,602 (3,034) | −20,511 (2,870) | −110 (27) | | 579 (42) |
| P-value | 0.0029 | 0.0030 | 0.0010 | 0.0010 | 0.0064 | | 0.0010 |
| Δ to reference combination (%) | −48.2 (0.63) | −47.9 (0.62) | −62.6 (0.87) | −62.8 (0.86) | −38.5 (1.44) | | −62.8 (0.86) |
| P-value | < 0.001 | < 0.001 | < 0.001 | < 0.001 | < 0.001 | | < 0.001 |
| Δ to least costly combination | 610,264 (89,890) | 631,061.5 (87,972) | −20,798 (2,967) | −19,788 (2810) | −62.0 (23.2) | | −947 (135) |
| P-value | 0.0007 | 0.0007 | 0.0010 | 0.0010 | 0.033 | | 0.0010 |
| Δ to least costly combination (%) | 144 (1.7) | 162 (1.8) | −61.7 (0.88) | −61.9 (0.88) | −26.1 (1.6) | | −61.9 (0.88) |
| P-value | < 0.001 | < 0.001 | < 0.001 | < 0.001 | 0.001 | | < 0.001 |
| ***4-months & 6-months all oral (shortest combination)*** | 1,034,617 (82,482) | 1,021,778 (81,583) | 12,838 (924) | 12,087 (870) | 173 (12) | | 579 (42) |
| Δ to reference combination | −1,275,158 (184,003) | −1,278,486 (180,337.5) | 3,328 (4,228) | 3,134 (4,001) | 43 (35) | | 150 (192) |
| P-value | 0.0011 | 0.0011 | 0.45 | 0.46 | 0.25 | | 0.46 |
| Δ to reference combination (%) | −63.6 (0.65) | −64.9 (0.64) | 9.7 (0.38) | 9.7 (0.38) | 16 (1.1) | | 9.7 (0.38) |
| P-value | < 0.001 | < 0.001 | < 0.001 | < 0.001 | < 0.001 | | < 0.001 |
| Δ to least costly combination | 352,696 (36,082) | 325,269 (33,230) | 27,427 (2,852) | 25,996 (2,703) | 187 (20) | | 1,245 (129) |
| P-value | 0.0005 | 0.0005 | 0.0005 | 0.0005 | 0.0005 | | 0.0005 |
| Δ to least costly combination (%) | 491 (18.7) | 496 (18.9) | 441 (16.8 | 441.2268 (16.8 | 385 (15) | | 441 (17) |
| P-value | < 0.001 | < 0.001 | < 0.001 | < 0.001 | < 0.001 | | < 0.001 |
|  | | | | | | | |
| **Import cost comparison between regimen combinations** | | | | | | | |
| ***6-months / 6-months all-oral (least-costly-to-import combination)*** | 732,532 (62,998) | 723,826 (62,387) | 8,706 (638) | 8,230 (603) | 82.0 (6.2) | | 394 (29) |
| Δ to reference combination | −1,269,064 (186,358) | −1,243330 (18,3437) | −25,735 (2,960) | −24,367 (2,801) | −201 (25) | | −1167 (134) |
| P-value | 0.0010 | 0.0010 | 0.0006 | 0.0006 | 0.0007 | | 0.0006 |
| Δ to reference combination (%) | −63.4 (0.084) | −63.2 (0.078) | −74.6 (0.50) | −74.7 (0.50) | −71.0 (0.45) | | −74.7 (0.50) |
| P-value | < 0.001 | < 0.001 | < 0.001 | < 0.001 | < 0.001 | | < 0.001 |
| ***4-months / 20-months with an injectable antibiotic (costliest-to-import combination)*** | 2,121,287 (178,238) | 2,016,458 (169,229) | 104,829 (9,013) | 99,443 (8,552) | 625 (52) | | 4,761 (409) |
| Δ to reference combination | 119,690 (250,059) | 49,302 (241,652) | 70,388 (9,465) | 66,846 (8,979) | 342 (57) | | 3,200 (430) |
| P-value | 0.645 | 0.84 | 0.0008 | 0.0008 | 0.0012 | | 0.0008 |
| Δ to reference combination (%) | 6.09 (0.55) | 2.62 (0.56) | 204 (0.92) | 205 (0.94) | 121 (0.43) | | 205 (0.94) |
| P-value | 0.0004 | 0.0094 | < 0.001 | < 0.001 | < 0.001 | | < 0.001 |
| Δ to least-costly-to-import combination | 1,388,755 (189,043) | 1,292,632 (180,363) | 96,123 (9,036) | 91,213 (8,573) | 543 (52) | | 4,367 (410) |
| P-value | 0.0007 | 0.0008 | 0.0004 | 0.0004 | 0.0004 | | 0.0004 |
| Δ to least-costly-to-import combination (%) | 190 (0.84) | 179 (0.93) | 1,101 (29) | 1,105.5 (28) | 661 (11) | | 1106 (28) |
| P-value | < 0.001 | < 0.001 | < 0.001 | < 0.001 | < 0.001 | | < 0.001 |

Δ = difference in 2016–20 means, (  ) = standard deviation, TB = tuberculosis, DS = drug-susceptible, MDR = multidrug-resistant. ^a^As percentage difference does not vary between years, no standard deviation and P-value are reported. Air freight costs were due to international shipping by plane from Amsterdam airport to Tashkent airport. Customs-related import costs include costs of a customs agent assisting with the declaration of the imported medical supplies and costs for storage at Tashkent airport during the declaration process. Land freight costs were due to national transport by truck from Tashkent airport to a central storage in Karakalpakstan’s capital Nukus. The TB drugs and dosing used in these regimens are provided in **Table 1**.
